# Comparative Analysis of Normoxia- and Hypoxia-Modified Extracellular Vesicle Therapy in Function, Perfusion, and Collateralization in Chronically Ischemic Myocardium

**DOI:** 10.3390/ijms24032076

**Published:** 2023-01-20

**Authors:** Sharif A. Sabe, Cynthia M. Xu, Brittany A. Potz, Akshay Malhotra, Mohamed Sabra, Dwight D. Harris, Mark Broadwin, M. Ruhul Abid, Frank W. Sellke

**Affiliations:** Division of Cardiothoracic Surgery, Department of Surgery, Cardiovascular Research Center, Rhode Island Hospital, Alpert Medical School of Brown University, Providence, RI 02903, USA

**Keywords:** extracellular vesicles, hypoxia-modified, myocardial ischemia, coronary microcirculation

## Abstract

We have previously shown that normoxia serum-starved extracellular vesicle (EV) therapy improves myocardial function, perfusion, and angiogenesis in a swine model of chronic myocardial ischemia. Hypoxia-modified EVs have increased abundance of anti-oxidant, pro-angiogenic, and pro-survival proteins. The purpose of this study is to investigate the differential effects of normoxia serum-starved EVs and hypoxia-modified EVs on myocardial function, perfusion, and microvascular density in chronically ischemic myocardium. Yorkshire swine underwent placement of an ameroid constrictor to the left circumflex artery to induce chronic myocardial ischemia. Two weeks later, the pigs underwent intramyocardial injection of either normoxia serum-starved EVs (NOR, *n* = 10) or hypoxia-modified EVs (HYP, *n* = 7). Five weeks later, pigs were euthanized, and ischemic myocardium was harvested. Hypoxia EV treatment was associated with improved contractility compared to NOR, as well as improved capillary density, without changes in arteriolar density. There were trends towards improved perfusion at rest and during pacing in the HYP group compared to NOR. Ischemic myocardium in the HYP group had increased pro-angiogenic Akt and ERK signaling and decreased expression of anti-angiogenic markers compared to the NOR group. In the setting of chronic myocardial ischemia, hypoxia-modified EVs may enhance contractility, capillary density, and angiogenic signaling pathways compared to normoxia serum-starved EVs.

## 1. Introduction

Therapeutic options in patients with chronic coronary artery disease (CAD) who are poor candidates for percutaneous or surgical revascularization remain limited [1]. CAD, caused by atherosclerotic plaque accumulation, can limit perfusion to myocardial tissue thereby contributing to tissue ischemia [2]. Patients left untreated in this setting are subject to chronic myocardial ischemic injury, which can result in adverse remodeling and heart failure [3]. Current pharmacological therapies are directed towards the mitigation of risk factors such as hypertension and hyperlipemia, and more direct therapies include catheter-based and surgical revascularization strategies which restore myocardial perfusion, thereby preventing progressive ischemic injury [4]. However, patients with advanced and diffuse CAD who have failed or are not candidates for revascularization have limited therapeutic options [1,4]. Therefore, the development of medical therapies for patients with chronic coronary diseases has been an area of active investigation.

Stem-cell-based therapies are an area of promising investigation in the treatment of chronic coronary disease; however, clinical trials have typically shown only modest and transient benefits [5]. Within the wide umbrella of stem-cell-based therapies, human bone mesenchymal stem-cell (HBMSC) derived extracellular vesicles (EVs) may play an important role in the development of therapeutic strategies for chronic coronary diseases [5,6]. EVs are small lipid-bound bioactive molecules containing growth factors, kinase receptors, and non-coding RNAs, and mediate the paracrine effects of mesenchymal stem-cells (MSC) [5]. When compared to their MSC progenitors, EVs have the advantages of decreased immunogenicity, higher safety profiles, and the ability to cross biological barriers [5]. Our recent studies using a swine model of chronic myocardial ischemia demonstrate that intramyocardial injection of EVs derived from normoxia, serum-starved HBMSC, improves myocardial function, perfusion, angiogenesis, and angiogenic signaling pathways compared to no treatment [7,8].

Importantly, the contents of EVs can be modified depending on the conditions the progenitor HBMSCs are subjected to. Specifically, hypoxia-preconditioning of MSC has been shown to enrich EV contents with proangiogenic growth factors and microRNAs compared to normoxia conditions [9]. Small animal models of acute myocardial infarction have shown that exosomes derived from hypoxia-treated MSCs may have cardioprotective effects compared to those derived from normoxia-treated MSCs [10,11]. However, the role of hypoxia-modified compared to normoxia EVs in the setting of chronic myocardial ischemia has not been previously investigated. Additionally, despite some studies in small animal models of acute myocardial infarction, there is a lack of data on the role of hypoxia-modified EVs in the treatment of myocardial ischemia in a more clinically relevant large animal model. Therefore, the purpose of this study is to investigate the differential effects of normoxia serum-starved EVs (NOR) and hypoxia-modified EVs (HYP) on myocardial function, coronary perfusion, and microvascular density in a swine model of chronic myocardial ischemia.

## 2. Results

### 2.1. Hemodynamic Parameters

**Figure 1:**
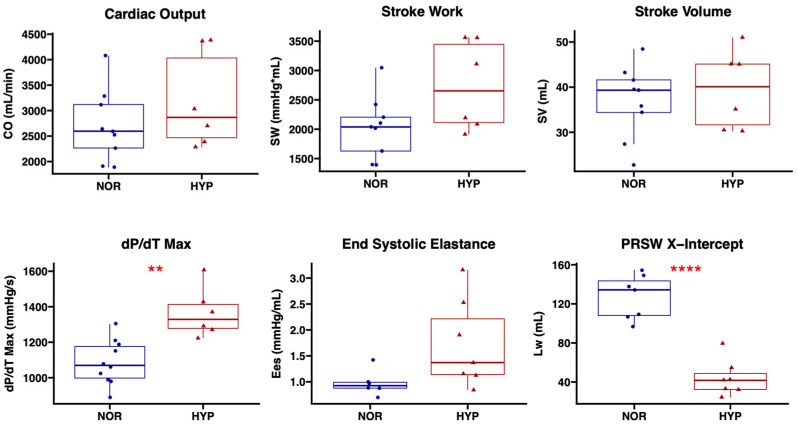
Intramyocardial injection of hypoxia-modified EVs increases left ventricular contractility compared to the injection of normoxia serum-stressed EVs. Swine treated with 50 µg hypoxia-modified (HYP, *n* = 7) EVs had improved contractility as measured by dP/dt_max_ and the x-intercept (Lw) of the pre-load recruitable stroke work relationship (PRSW) compared to swine treated with 50 µg normoxia serum-starved EVs (NOR, *n* = 10). There was a trend towards improved stroke work and end-systolic elastance in the HYP group compared to NOR, without changes in cardiac output or stroke volume. Upper and lower borders of box represent upper and lower quartiles; middle horizontal line represents median; upper and lower whiskers represent maximum and minimum values. CO, cardiac output; SW, stroke work; SV, stroke volume; dP/dT, change in pressure over change in time; Ees, end-systolic elastance. ** *p* < 0.01, **** *p* < 0.0001.

Cardiac hemodynamic parameters were collected using a pressure-volume catheter placed within the left ventricular apex, in order to determine the effect of EVs on cardiac function in the setting of chronic myocardial ischemia. There were no significant differences in stroke volume (*p* = 0.58) or cardiac output (*p* = 0.31) between the NOR and HYP groups. There was a trend towards improved stroke work in the HYP group compared to NOR (*p* = 0.081). There was improved left ventricular contractility in the HYP group as measured by the load-dependent dP/dt_max_ (*p* = 0.003) as well as the load-independent x-intercept (Lw) of the pre-load recruitable stroke work relationship (PRSW, *p* < 0.001). There was a trend towards improved end-systolic elastance (*p* = 0.060), another load-independent measure of contractility, in the HYP group compared to NOR (Figure 1).

### 2.2. Myocardial Perfusion

**Figure 2:**
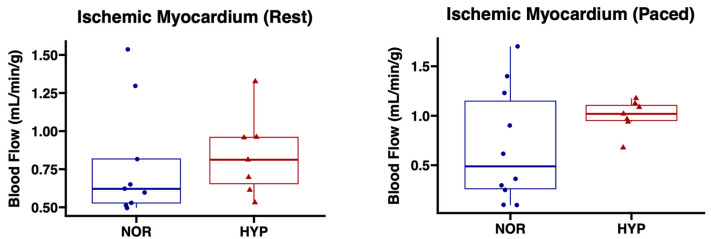
Perfusion to chronically ischemic myocardium was not significantly changed with hypoxia-modified EVs compared to normoxia EVs. Swine treated with hypoxia-modified (HYP, *n* = 7) EVs had nonsignificant trends towards increased perfusion to chronically ischemic myocardial territory compared to swine treated with normoxia serum-starved EVs (NOR, *n* = 10). Upper and lower borders of box represent upper and lower quartiles; middle horizontal line represents median; upper and lower whiskers represent maximum and minimum values.

There were nonsignificant trends towards increased absolute blood flow to chronically ischemic myocardium both at rest (*p* = 0.35) and during pacing (*p* = 0.15) in the HYP group compared to NOR (Figure 2).

### 2.3. Microvessel Density

**Figure 3:**
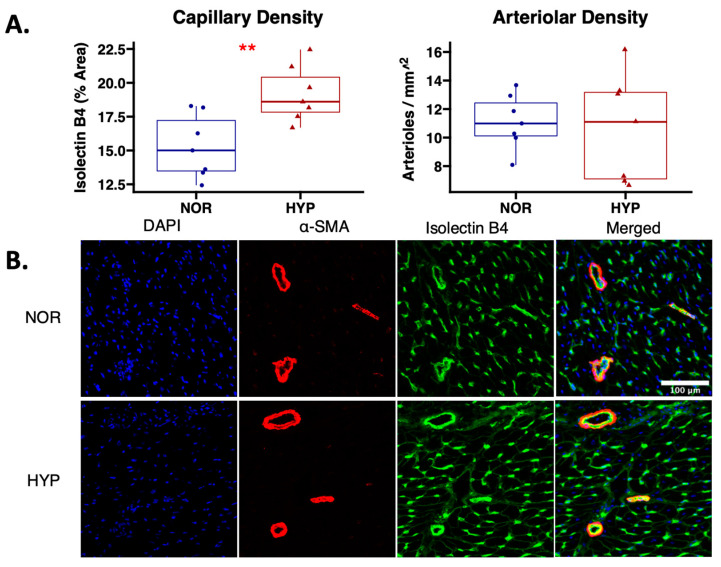
Hypoxia-modified EVs increased capillary density to chronically ischemic myocardium compared to normoxia EVs. (**A**) Swine treated with hypoxia-modified (HYP, *n* = 7) EVs had improved capillary density as measured by isolectin B4 immunofluorescent staining compared to treatment with normoxia serum-starved EVs, without changes in arteriolar density as measured by α-smooth muscle actin staining. The upper and lower borders of the box represent upper and lower quartiles; the middle horizontal line represents the median; the upper and lower whiskers represent maximum and minimum values. ** *p* < 0.01. (**B**) Representative images (20×) of ischemic myocardial tissue stained with α-smooth muscle actin (α-SMA) for arteriolar density (red), isolectin B4 for capillary density (green), and 4′,6-diamidino-2-phenylindole (DAPI) for nuclei staining (blue).

Capillary density, as measured by isolectin B4 immunofluorescent staining, was increased in chronically ischemic myocardium of swine treated with hypoxia-modified EVs compared to normoxia-serum starved EVs (*p* = 0.007). There were no changes in arteriolar density, as measured by α-smooth muscle actin immunofluorescent staining between groups (*p* = 0.77) (Figure 3).

### 2.4. Angiogenic Signaling

#### 2.4.1. Pro-Angiogenic Signaling

In chronically ischemic myocardium treated with hypoxia-modified EVs, immunoblot experiments showed an increased expression of p-Akt (*p* = 0.002) and the p-Akt to Akt ratio (*p* < 0.001) compared to the NOR group, without changes in total Akt (*p* = 0.26). There was increased expression of total ERK1/2 (*p* = 0.005) in the HYP group, without changes in the expression of p-ERK1/2 (*p* = 0.85) or the p-ERK1/2 to ERK1/2 ratio (*p* = 0.80). There were no significant differences in the expression of p-eNOS (*p* = 0.41) or total eNOS (*p* = 0.15), though there was a strong trend towards a decreased p-eNOS to eNOS ratio (*p* = 0.055) in the HYP group compared to NOR. There was an increased expression of VE-cadherin (*p* < 0.001) in the HYP group compared to NOR.

#### 2.4.2. Anti-Angiogenic Signaling

**Figure 4:**
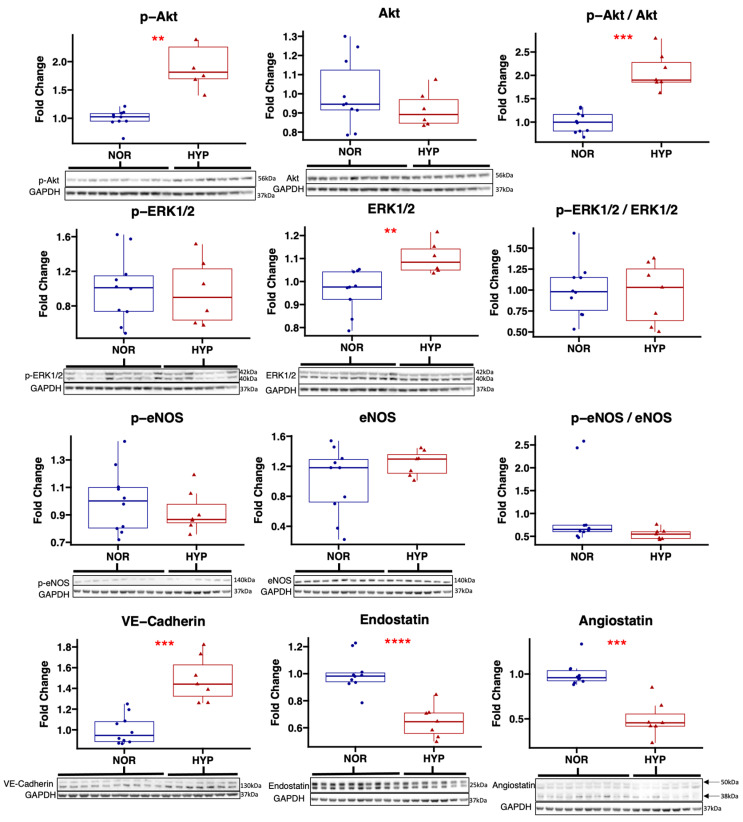
Hypoxia-modified EV treatment increased pro-angiogenic signaling and decreased anti-angiogenic signaling in chronically ischemic myocardium compared to normoxia EV treatment. Immunoblot results from total protein lysates of chronically ischemic myocardium in swine treated with hypoxia-modified EVs (HYP, *n* = 7) compared to normoxia serum-starved EVs (NOR, *n* = 10) are shown for pro-angiogenic markers Akt, phosphorylated (p-) Akt, and the ratio of p-Akt to Akt; extracellular signal-regulated kinase 1/2 (ERK1/2), p-ERK1/2, and the ratio of p-ERK 1/2 to ERK1/2; endothelial nitric oxide synthase (eNOS), p-eNOS, and the ratio of p-eNOS and eNOS; and vascular endothelial cadherin (VE-Cadherin). Immunoblot results are also shown for anti-angiogenic markers angiostatin and endostatin. Upper and lower borders of box represent upper and lower quartiles; middle horizontal line represents median; upper and lower whiskers represent maximum and minimum values. ** *p* < 0.01, *** *p* < 0.001, **** *p* < 0.0001.

There was a decreased expression of anti-angiogenic signaling markers endostatin (*p* < 0.001) and angiostatin (*p* < 0.001) in the HYP group compared to NOR (Figure 4).

## 3. Discussion

In the present study, we found that in our large animal model of chronic myocardial ischemia, intramyocardial injection of hypoxia-modified EVs, when compared to normoxia serum-starved EVs, is associated with (1) improved cardiac contractility as measured by dP/dt_max_ and Lw derived from the PRSW relationship, (2) increased capillary density in ischemic myocardial tissue with trends towards improved perfusion, (3) increased proangiogenic signaling markers including Akt, ERK1/2, and VE-Cadherin, and (4) decreased anti-angiogenic signaling markers endostatin and angiostatin.

Our group has previously investigated the role of intramyocardial delivery of normoxia serum-starved EVs in the setting of chronic myocardial expression. EV therapy in this setting improved myocardial function, remodeling, perfusion to ischemic territory, capillary, and arteriolar collateralization, and proangiogenic signaling pathways [7,12,13]. Previous studies have demonstrated that hypoxia-modified EVs may augment the benefits of EV therapy in the setting of myocardial ischemia. In small animal models of acute myocardial infarction, exosomes derived from hypoxia-treated MSCs improved survival, infarct size, cardiac function, and angiogenesis compared to exosomes derived under normoxic conditions [10,11]. However, the differential effect of hypoxia-modified versus normoxia EVs in the treatment of chronic myocardial ischemia was previously unknown. This study, therefore, provides novel findings on the effects of hypoxia-modified EVs compared to normoxia EVs in a clinically relevant large animal model of chronic myocardial ischemia.

Hypoxia-modified EV therapy was associated with improved measures of contractility compared to normoxia EVs. dP/dt_max_ is a measure of the maximal rate of left ventricular pressure and an indicator of cardiac contractility, though it is limited by preload-dependence. Inferior vena cava occlusions, as performed in this model, allow for more reliable pre-load independent measures of contractility, including end-systolic elastance and the PRSW relationship. We found a strong trend towards an increased end-systolic elastance, indicating improved contractility. The volume axis intercept of the PRSW relationship, Lw, is an important indicator of cardiac contractility, with reduced Lw reflecting increased contractility, and the decreased Lw in hypoxia-modified compared to normoxia EV-treated pigs is reflective of improved contractility.

Furthermore, improvements in capillary density with hypoxia-modified EVs in our model are consistent with previous findings in small animal acute infarction studies comparing hypoxia-modified and normoxia EVs [10]. Improvement in capillary density may be secondary to increased Akt and ERK1/2 signaling. Normoxia EVs have been previously shown to activate Akt and ERK signaling in ischemic myocardium [7,14]. Li and others found that hypoxia-preconditioned MSCs had increased expression and activation of Akt compared to MSCs cultured in normoxia conditions [15]. These alterations may contribute to improved angiogenic therapeutic effects in our model. The increased expression of VE-cadherin, an important adhesion molecular in angiogenesis, in hypoxia-modified EVs compared to normoxia EVs may also contribute to increased collateralization. Furthermore, hypoxia-modified EVs may have increased effects on capillary collateralization compared to normoxia EVs due to decreased anti-angiogenic signaling. Both angiostatin and endostatin are potent inhibitors of angiogenic growth and endothelium-dependent vasodilation in coronary vasculature [16,17,18,19]. Normoxia EVs may play a role in decreasing these anti-angiogenic markers in the setting of chronic myocardial ischemia [20], and the findings in the present study suggest that hypoxia-modified EVs may better target anti-angiogenic signaling to improve collateralization in the setting of myocardial ischemia. Though there were trends towards improved perfusion at rest and during pacing with hypoxia-modified EV therapy compared to the normoxia EVs, these trends did not reach statistical significance. This may be secondary to the low sample size in this large animal model, or perhaps the increased capillary density was not adequate enough to improve myocardial perfusion. Nonetheless, improved capillary density has important clinical implications, particularly in the setting of acute insults to myocardial perfusion.

An important consideration in comparing the in-vivo effects of normoxia and hypoxia-modified EVs is how their differential contents affect their therapeutic potential. EVs are known to contain growth factors, kinase receptors, and non-coding RNAs that can promote regenerative and angiogenic effects in myocardial tissue [5]. Hypoxia preconditioning of HBMSC results in an increased expression of pro-angiogenic factors within extracellular vesicles, including pro-angiogenic microRNAs. We have ongoing studies comparing the proteomic profile of normoxia and hypoxia-modified EVs to provide additional mechanistic evidence of how hypoxia-modified EVs may exert their benefits in the setting of chronic myocardial ischemia. An additional consideration of intramyocardial HBMSC-EV therapy is what cell types are differentially targeted for EV cargo uptake. Zwi-Dantsis and others have shown that, using fluorescence-based tagging techniques, EVs derived from human cardiomyocytes are taken up by myocardial endothelial cells, cardiomyocytes, and fibroblasts, with preferential uptake by endothelial cells [21]. However, data are scant in this area investigating EVs derived from other cell types, including HBMSC. Given the effects of HBMSC-EV on capillary collateralization and pro-angiogenic signaling, uptake by endothelial cells would be expected; however, further investigation into myocardial cell-specific uptake of HBMSC-EV would provide more definitive clarity.

There are several limitations in this study to consider. One limitation is the low sample size in this large animal model, which may have resulted in an underpowered analysis, particularly with our perfusion data. An additional limitation is the single time point of measurement in functional and molecular parameters. Our data collection occurred five weeks following intramyocardial EV injection, and therefore our analysis was limited to downstream effects of EVs, which are typically short-lived and likely to regulate several molecular pathways in the early period which we had not captured in the current model. However, the findings in this study and in others may help guide future studies in studying the temporal effects of intramyocardial EV injection over the course of hours to days and to weeks. Finally, intramyocardial delivery of EVs is limited in clinical practicality. Future studies investigating methods of delivering EVs to myocardial tissue through less invasive routes, such as intravenous or intracoronary, would help translate the benefits of EVs to clinical practice.

## 4. Materials and Methods

The data that support the findings of this study are available from the corresponding author upon reasonable request.

### 4.1. HBMSC Preconditioning and EV Isolation

Human bone marrow-derived stem-cells (HBMSC) (Lonza, Allendale, NJ, USA) were cultured according to Lonza recommendations in growth media (MSCGM Bulletkit PT-3001; Lonza, Allendale, NJ, USA). Cells were grown to 80% confluence and were passaged to passage 7 and were designated for either normoxia serum-starved or hypoxia-modification pre-conditioning. In cells designated to normoxia serum-starved pre-conditioning, the growth media was removed, cells were washed with PBS, media was replaced with serum-free Roswell Park Memorial Institute (RPMI) medium, and placed in a standard incubator at 37 °C with 5% CO_2_. After 24 h of incubation, the serum-free media was collected and centrifuged at 2000× *g* at 4 °C to remove cellular debris. EVs were then isolated by ultracentrifugation at 100,000× *g* for 70 min, washed with PBS at 100,000× *g* for 70 min, and then resuspended in 1% dimethyl sulfoxide in PBS. Samples were snap-frozen in liquid nitrogen and stored at −80 °C. In cells designated for hypoxia modification, the growth media was replaced with fresh MSCGM media, and cells were placed in an airtight, humidified hypoxia chamber (Billups-Rothenberg, MIC-101) containing 95% nitrogen and 5% carbon dioxide. Hypoxia was induced by placing the cells in the chamber and then connecting the hypoxia chamber inflow cannula to a gas tank containing 95% nitrogen and 5% carbon dioxide at a flow rate of 20 L/minute with the outflow cannula open for seven minutes to wash out oxygen, then clamping shut the outflow and inflow cannulas simultaneously. The cells were incubated in the hypoxia chamber at 37 °C for 24 h. Then the hypoxia chambers were opened, the media was collected, and EVs were isolated as described earlier. Protein quantification was performed using a radioimmunoprecipitation assay (Kit 23225, Thermo Fisher Scientific, Waltham, MA, USA) to quantify a standard dose for intramyocardial injection. Characterization was determined by immunoblotting with incubation of 1:1000 dilutions of primary antibodies to CD81 (Cell Signaling, 52892S), CD9 (Cell Signaling, 13403S), Alix (Cell Signaling, 92880S), and albumin (Cell Signaling, 4929S), followed by incubation with anti-rabbit secondary antibody (Cell Signaling, 7074) (complete antibody information listed in Table S1), followed by chemiluminescent detection (ThermoFisher Scientific, Waltham, MA, USA), and capture with a digital camera system (Bio-Rad ChemiDoc MP, Life Science, Hercules, CA, USA). Characterization was further performed using nanoparticle tracking analysis and transmission electron microscopy at 80 kV (FEI Morgagni 268) after fixation in 2% paraformaldehyde for 20 min and negative staining in 4% uranyl acetate (Figure S1). A total of 50 µg of EVs was thawed and suspended in 2 mL 0.9% sterile saline on the day of administration.

### 4.2. Animal Model

All experiments were approved by the Institutional Animal Care and Use Committee of the Rhode Island Hospital (#504621), and animals were cared for in coordination with veterinary technicians at Rhode Island Hospital in compliance with the Principles of Laboratory Animal Care formulated by the National Society of Medical Research and the Guide for the Care and Use of Laboratory Animals.

Seventeen Yorkshire swine at age 11 weeks underwent thoracotomy for placement of an ameroid constrictor (Research Instruments SW, Escondido, CA, USA) around the proximal left circumflex artery to induce chronic myocardial ischemia. A second thoracotomy procedure was performed two weeks later for intramyocardial injection of either normoxia serum-starved EVs (NOR, *n* = 10) or hypoxia-modified EVs (HYP, *n* = 7). Five weeks later, animals were euthanized and tissue was harvested for analysis.

### 4.3. Ameroid Constrictor Placement

Anesthesia for procedures, perioperative analgesia, and prophylaxis were carried out as described previously [7]. A left mini-thoracotomy was made, the pericardium was opened, and the left atrium was retracted to expose the left circumflex artery, which was then isolated near its takeoff from the left main coronary artery. The pig was systemically heparinized, and the left circumflex artery was occluded for two minutes as confirmed by ST and/or T wave changes on telemetry monitoring. During occlusion, 5 mL of gold-labeled microspheres (BioPal, Worcester, MA, USA) were injected into the left atrium. An ameroid constrictor (Research Instruments SW, Escondido, CA, USA) was sized according to the left circumflex artery diameter and placed around the artery. Topical nitroglycerin was applied over the vessel as needed to reverse vasospasm. The incision was closed, and the pigs were recovered in a monitored setting.

### 4.4. Intramyocardial Injection of EVs

Two weeks after ameroid constrictor placement, a left-mini thoracotomy incision was made one rib space below the prior incision. The pericardium was opened, and the at-risk left ventricular myocardium was identified as inferior to the previously placed-ameroid constrictor. Animals then underwent intramyocardial injection with 50 µg of either normoxia serum stressed EVs or hypoxia-modified EVs, using a 27-gauge needle inserted 0.5 cm into the myocardium at eight locations, as determined by tracking branches off the left circumflex artery and injecting into myocardium supplied by these branches approximately 2–3 cm apart. The wound was then closed, and pigs were recovered in a monitored setting.

### 4.5. Terminal Harvest Procedure

Five weeks after the EV injection, pigs underwent a terminal harvest procedure. Femoral artery access was obtained. A median sternotomy was performed and the heart was exposed. For blood flow analyses, isotope-labeled microspheres were injected into the left atrium while 10 mL of blood was withdrawn from a femoral artery catheter. For hemodynamic measurements, a pressure-volume catheter (Transonic, Ithaca, NY, USA) was placed into the apex of the left ventricle. Finally, the heart was excised, and myocardial tissue was divided into 16 segments based on location with respect to the left anterior descending and left circumflex arteries. Myocardial tissue segments were air-dried for microsphere analysis or snap-frozen in liquid nitrogen for immunoblot analysis and frozen sectioning. The proximal circumflex artery in the area of the ameroid constrictor was inspected to confirm occlusion.

### 4.6. Hemodynamic Measurements

During the harvesting procedure, a pressure-volume catheter (Transonic, Ithaca, NY, USA) was inserted directly into the left ventricular apex for cardiac hemodynamic measurements. Load-dependent data were collected during breath holds to minimize respiratory variation effects. Load-independent data were collected during breath holds by occluding the inferior vena cava with a vessel loop. Hemodynamic recordings were analyzed with LabChart software (ADInstruments, Colorado Springs, CO, USA).

### 4.7. Myocardial Perfusion Measurements

Myocardial perfusion was measured using isotope-labeled microspheres (Biophysics Assay Laboratory, Worcester, MA, USA). During the ameroid procedure, the left circumflex was occluded temporarily and 5 mL of gold-labeled microspheres were injected into the left atrium to determine the territory of the left ventricle supplied by the left circumflex artery. During the harvesting procedure, 5 mL of lutetium-labeled and samarium-labeled microspheres were injected into the left atrium at rest and during pacing, respectively, while simultaneously withdrawing 10 mL of blood from the femoral artery at a reference rate of 6.67 mL/min using a withdrawal pump (Harvard Apparatus, Holliston, MA, USA). Left ventricular myocardial samples from 10 sections based on proximity of location to the left anterior descending and left circumflex artery were weighed and dried at 70 °C. Tissue samples and blood were sent to the Biophysics Assay laboratory for microsphere density measurements. Blood flow was calculated using the following equation: tissue blood flow = [reference blood flow (mL/min)/tissue weight (g)] × [tissue microsphere count/reference blood microsphere count].

### 4.8. Microvessel Density Studies

Microvessel density was determined by immunofluorescent staining, which was performed as previously described [22]. Briefly, frozen sections were thawed, fixed with 10% paraformaldehyde, blocked in 3% bovine serum albumin, and incubated overnight at 4 °C with primary antibody to α-smooth muscle actin (Abcam, Cambridge, UK) for arteriolar staining and isolectin B4 conjugated to Alexa Fluor 647 for capillary staining (Thermo Fisher Scientific, Waltham, MA, USA). Slides were rinsed with PBS and incubated with an anti-mouse secondary antibody conjugated to Alexa Fluor 594 (Cell Signaling, Danvers, MA, USA) at room temperature for one hour. Slides were rinsed, and DAPI was applied for five minutes, then rinsed again and mounted. Images were analyzed at 20× magnification with an Olympus VS200 Slide Scanner (Olympus Corporation, Tokyo, Japan). Image analysis was performed with QuPath software [23]. Capillary density was determined by thresholding positive isolectin B4 staining and determining the percent of tissue area stained. The arteriolar count was determined by thresholding positive SMA staining and determining the number of objects with a minimum size of 100 µm^2^ per area of tissue sections.

### 4.9. Immunoblotting Studies

Ischemic myocardial tissue was lysed in radioimmunoprecipitation assay buffer (Boston Bioproducts, Milford, MA, USA). Total protein (40 µg) was fractionated onto a 4–12% Bis-Tris gel (ThermoFisher Scientific, Waltham, MA, USA), and membranes were incubated overnight at 4 °C with 1:1000 dilutions of individual rabbit polyclonal primary antibodies to Akt (CS9272), phosphorylated (Ser473) Akt (p-Akt) (CS4060), extracellular signal-regulated kinase 1/2 (ERK1/2) (CS4695), phosphorylated ERK1/2 (p-ERK1/2) (CS4370), endothelial nitric oxide synthase (eNOS) (CS32027), phosphorylated (Ser1177) eNOS (p-eNOS) (CS9571), vascular endothelial cadherin (VE-Cadherin) (CS2500) (Cell Signaling, Danvers, MA, USA), endostatin (ab207162) (Abcam, Cambridge, UK), and 1:500 dilution of primary antibody to angiostatin (ab2904) (Abcam, Cambridge, UK) (complete antibody information listed in Table S1). All membranes were probed with glyceraldehyde 3-phosphate dehydrogenase (GAPDH) (CS97166) (Cell Signaling, Danvers, MA, USA) to correct for loading errors. Membranes were incubated with anti-mouse or anti-rabbit secondary antibodies (Cell Signaling, Danvers, MA) at room temperature for one hour, processed for chemiluminescent detection (ThermoFisher Scientific, Waltham, MA, USA), and captured with a digital camera system (Bio-Rad ChemiDoc MP, Life Science, Hercules, CA, USA). Densitometric analysis of band intensity was performed using NIH Image J software.

### 4.10. Data Analysis

All data were tested for normality with a Shapiro-Wilk test. Normal data were statistically analyzed with a student’s *t* test after removing outliers greater than 2 standard deviations from the mean, and non-normal data were analyzed with a Wilcoxon rank-sum test including all outliers. All data analysis was performed using R software. Probability values < 0.05 were considered statistically significant.

## 5. Conclusions

In the setting of chronic myocardial ischemia, hypoxia-modified EV therapy, when compared to normoxia EV therapy, is associated with improved cardiac contractility and improved capillary collateralization via increased Akt and ERK signaling, and decreased anti-angiogenic signaling.

## Data Availability

The data that support the findings of this study are available from the corresponding author upon reasonable request.

## Supplementary Materials

The supporting information can be downloaded at: https://www.mdpi.com/article/10.3390/ijms24032076/s1.
