# Supplementary material for: Comparative Analysis of Normoxia- and Hypoxia-Modified Extracellular Vesicle Therapy in Function, Perfusion, and Collateralization in Chronically Ischemic Myocardium
Source: Int J Mol Sci. 2023 Jan 20;24(3):2076. doi: 10.3390/ijms24032076 (PMC9916784; doi:10.3390/ijms24032076)
Supplement: Supplementary file 1 [file ijms-24-02076-s001.zip › ijms-2168040-supplementary.pdf]

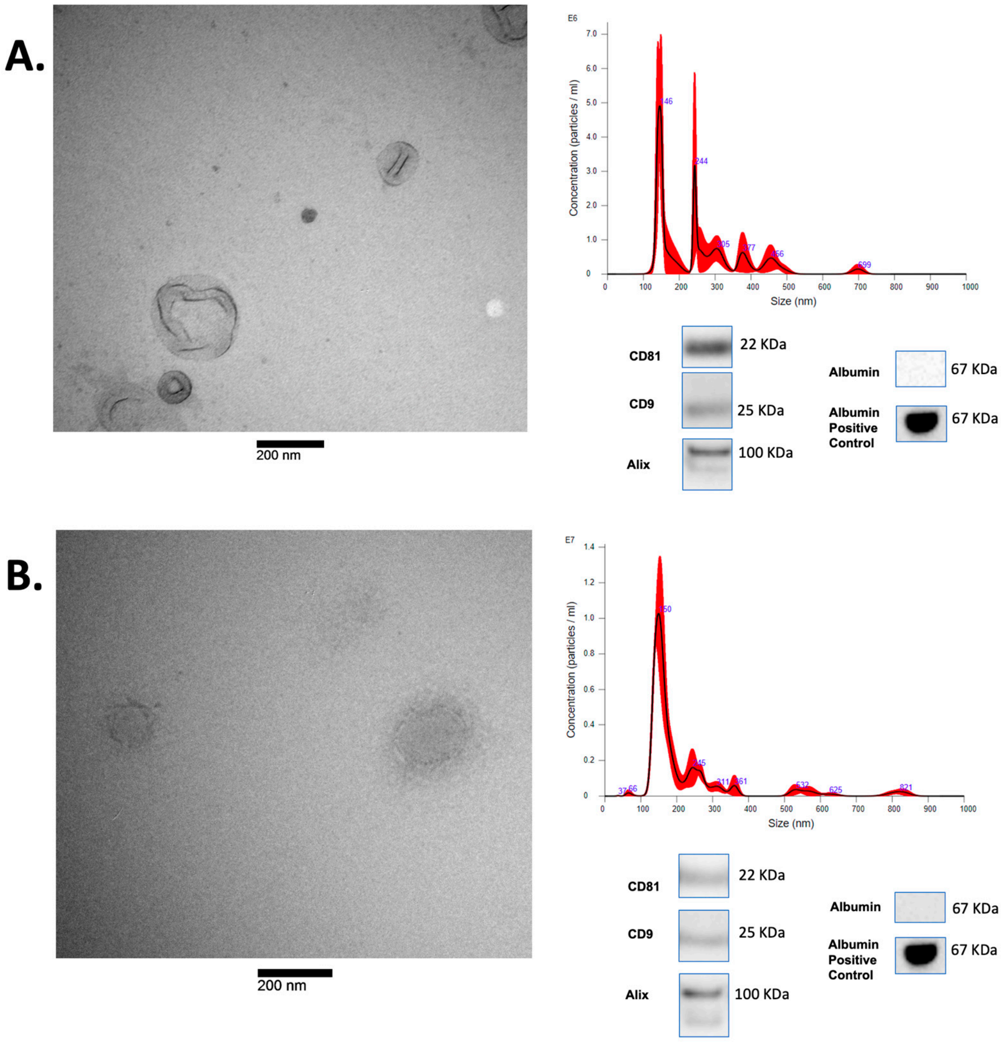

Figure S1. Characterization of HBMSC-EV. (**A**) Normoxia serum-starved EV characterization shown, including electron microscopy image of human bone marrow-derived mesenchymal stem-cell extracellular vesicle (HBMSC-EV) particle (magnification 54,800×), nanoparticle tracking analysis which determined mean particle size to be 252.7 nm ± 9.7 nm, and Western blot which identified typical HMBSC-EV transmembrane proteins CD81 and CD9, and cytosolic protein Alix. The lack of albumin demonstrates sample purity. (**B**) Hypoxia-modified EV characterization shown, including electron microscopy image of HBMSC-EV particle (magnification 54,800×), Nanoparticle tracking analysis which determined mean particle size to be 217.3 nm ± 10.9 nm, and Western blot which identified typical HMBSC-EV transmembrane proteins CD81 and CD9, and cytosolic protein Alix. The lack of albumin demonstrates sample purity.

Table S1. Antibody Information.

Antibody Name	Source Species	Dilution	Manufacturer Name	Manufacturer Location	Catalog Number
Akt	Rabbit	1:1000	Cell Signaling	Danvers, MA, USA	9272
Albumin	Rabbit	1:1000	Cell Signaling	Danvers, MA, USA	4929S
Alix	Rabbit	1:1000	Cell Signaling	Danvers, MA, USA	92880S
Angiostatin	Rabbit	1:500	Abcam	Cambridge, UK	2904
Anti-Rabbit IgG, HRP-linked	Goat	1:4000	Cell Signaling	Danvers, MA, USA	7074
CD81	Rabbit	1:1000	Cell Signaling	Danvers, MA, USA	52892S
CD9	Rabbit	1:1000	Cell Signaling	Danvers, MA, USA	13403S
Endostatin	Rabbit	1:1000	Abcam	Cambridge, UK	207162
eNOS	Rabbit	1:1000	Cell Signaling	Danvers, MA, USA	32027
ERK1/2	Rabbit	1:1000	Cell Signaling	Danvers, MA, USA	4695
GAPDH	Mouse	1:1000	Cell Signaling	Danvers, MA, USA	97166
p-Akt (Ser473)	Rabbit	1:1000	Cell Signaling	Danvers, MA, USA	4060
p-eNOS (Ser1177)	Rabbit	1:1000	Cell Signaling	Danvers, MA, USA	9571
p-ERK1/2 (Thr202/204)	Rabbit	1:1000	Cell Signaling	Danvers, MA, USA	4370
VE-Cadherin	Rabbit	1:1000	Cell Signaling	Danvers, MA, USA	2500

Antibodies used in this study are listed along with corresponding source species, dilution, manufacturer and catalog numbers. IgG, immunoglobulin G; HRP, horseradish peroxidase; CD, cluster of differentiation; eNOS; endothelial nitric oxide synthase; ERK, extracellular regulated kinase 1/2; GAPDH, glyceraldehyde-3-phosphate dehydrogenase; p-, phosphorylated; VE-Cadherin, vascular endothelial cadherin.
